# DNA methylation data-based molecular subtype classification and prediction in patients with gastric cancer

**DOI:** 10.1186/s12935-020-01253-4

**Published:** 2020-07-29

**Authors:** Qixin Lian, Bo Wang, Lijun Fan, Junqiang Sun, Guilai Wang, Jidong Zhang

**Affiliations:** 1 Oncology Department, First Affiliated Hospital of Jiamusi University, 154002 Qiqihar, Heilongjiang China; 2 Gastroenterology Department, The First Hospital of Qiqihar, The Affiliate Qiqihar Hospital of Southern Medical University, Longsha District, 30 of Park Road, Qiqihar, Heilongjiang 161005 China; 3 Radiotherapy and Chemotherapy, The First Hospital of Dandong, Liaoning, 118000 China; 4 General Surgery, The First Hospital of Qiqihar, The Affiliate Qiqihar Hospital of Southern Medical University, Longsha District, 30 of Park Road, Qiqihar, Heilongjiang 161005 China

## Abstract

**Background:**

Genetic and epigenetic alterations have been indicated to be closely correlated with the carcinogenesis, DNA methylation is one of most frequently occurring molecular behavior that take place early during this complicated process in gastric cancer (GC).

**Methods:**

In this study, 398 samples were collected from the cancer genome atlas (TCGA) database and were analyzed, so as to mine the specific DNA methylation sites that affected the prognosis for GC patients. Moreover, the 23,588 selected CpGs that were markedly correlated with patient prognosis were used for consistent clustering of the samples into 6 subgroups, and samples in each subgroup varied in terms of M, Stage, Grade, and Age. In addition, the levels of methylation sites in each subgroup were calculated, and 347 methylation sites (corresponding to 271 genes) were screened as the intrasubgroup specific methylation sites. Meanwhile, genes in the corresponding promoter regions that the above specific methylation sites were located were performed signaling pathway enrichment analysis.

**Results:**

The specific genes were enriched to the biological pathways that were reported to be closely correlated with GC; moreover, the subsequent transcription factor enrichment analysis discovered that, these genes were mainly enriched into the cell response to transcription factor B, regulation of MAPK signaling pathways, and regulation of cell proliferation and metastasis. Eventually, the prognosis prediction model for GC patients was constructed using the Random Forest Classifier model, and the training set and test set data were carried out independent verification and test.

**Conclusions:**

Such specific classification based on specific DNA methylation sites can well reflect the heterogeneity of GC tissues, which contributes to developing the individualized treatment and accurately predicting patient prognosis.

## Background

From the perspective of the world, gastric cancer has a higher incidence and ranks fourth [1]. Throughout China, gastric cancer has a higher incidence and mortality rate, ranking second among malignant tumors [2]. The number of deaths from malignant diseases is also the highest among malignant tumors of the digestive system [3]. Gastric cancer is a malignant tumor that originates from the gastric mucosa epithelium, due to changes in dietary structure, increased work pressure, and *H. pylori* infection, gastric cancer is becoming younger [4]. Radical surgery is currently the only possible cure for early gastric cancer, but because there are no obvious s in the early stage, it is often similar to the symptoms of chronic gastric diseases such as gastritis and gastric ulcer, and it is easy to be ignored [5]. Coupled with the low popularity of screening for gastric cancer in China, more than half of the patients were already in the middle and advanced stages at the time of diagnosis and lost the chance of radical surgery [6]. Surgical treatment combined with radiotherapy and chemotherapy have made some progress in the treatment of advanced gastric cancer, but the prognosis and quality of life of patients are still not ideal and need to be improved [7]. Therefore, further strengthening the research on the mechanism of gastric cancer occurrence and development, and looking for biological markers with stronger sensitivity and specificity, will help early detection and intervention of tumors, predict the prognosis of tumors, and develop more effective anti-tumor drugs. Important content in the prevention and treatment of gastric cancer.

As we know, the same cancer manifests differently in different individuals, but the same disease manifests different treatments [8]. Although the different stages of cancer reflect some characteristics of cancer, they can also help clinicians to develop treatment plans for specific stages. However, the individual differences due to changes in molecular level make the same pathological staging with the same treatment scheme get different treatment results [9, 10]. The root cause of this phenomenon is that clinicians do not know much about the molecular mechanisms of the occurrence, progression and metastasis of specific cancers in the human body, and cannot reach the stage of individualized treatment.

The method to cope with this problem is to use bioinformatics to conduct systematic research on a certain molecular level of cancer with the support of a large number of clinical samples to find the cause of cancer or find some genes caused by cancer changes in expression levels [11]. The purpose of this study was to identify molecular subtypes of gastric cancer at the gene expression level and methylation level using public data, find out the relationship between each cluster and clinical data, determine the unique molecular level characteristics of each cluster and establish corresponding The classifier obtains the label-like genes and classifiers for the classification on the sample training set, and then uses the test set to verify the classification effect of the label-like genes and classifiers. The tag-like genes and classifiers obtained in this way can perform category prediction on new samples and achieve the purpose of identifying cancer subtypes in new samples, so as to establish targeted treatment schemes for individuals in order to reduce cancer patient mortality and increase patients’ living standard goals.

## Methods

### TCGA data download and preprocessing

We used the TCGA GDC API to download the latest clinical follow-up information (2019.01.04), which contains a total of 398 samples (2 samples were paired normal counterparts named TCGA-xx-xxxx-11); RNA-Seq data was downloaded from TCGA GDC API, which contains a total of 450 samples (35 samples were paired normal counterparts named TCGA-xx-xxxx-11). UCSC Cancer Browser was used to download the Illumina Infinium Human Methylation 450 data included a total of 398 samples (2 samples were paired normal counterparts named TCGA-xx-xxxx-11). The exclusion criteria: NA was removed from all samples CpG sites with a value ratio exceeding 70%, while removing cross-reactive CpG sites in the genome according to the cross-reactive sites provided by Discovery of cross-reactive probes and polymorphic CpGs in the Illumina Infinium HumanMethylation450 microarray. Using the KNN method of R package impute to fill the methylation spectrum with missing values, and further remove the unstable genome methylation sites, that is, the CpGs and single nucleotide positions on the sex chromosome are removed Sites; 335,230 methylation sites were finally obtained.

### Random grouping of samples

First, 394 samples are equally divided into training and test set. The following criteria: 1. The samples were randomly assigned to the training and testing sets; 2. The age distribution, clinical stage, follow-up time, and proportion of patient deaths need to be similar in the two groups.

#### Survival analysis and molecular subtype screening of methylation sites in training set

A univariate Cox proportional hazards regression model was performed for each methylation site and survival data. Using the R package survival coxph function, p < 0.05 was selected as the threshold value. In the end, there were 23,588 significant prognostic difference sites, of which the most significant top 20 are shown in Table 1. Add Stage, Grade, and Age as covariates for CpG survival analysis: Through a univariate Cox model, select significant methylation sites for a multivariate Cox proportional hazard regression model, with M, Stage, Grade, and Age as covariates obtain significant multi-factor methylation sites. Take the intersection of the two to obtain 22,062 significant methylation sites. The result is Additional file 1: Table S1. Further, we used the R software package Consensus Cluster Plus to perform consistent clustering of methylation sites that were significant in both single and multiple factors to screen molecular subtypes. Euclidean distance is used to calculate the similarity distance between samples, and K-means is used for clustering.

**Table 1:** The most significant top 20 methylation sites

CpGs	p.value	HR	Low 95%CI	High 95%CI
cg11948421	2.94E − 08	442.2458396	51.34338135	3809.281304
cg08260760	8.75E − 08	2929.373453	157.3735659	54527.76507
cg16718891	1.95E − 07	1627.178489	100.4336939	26362.76462
cg01805784	2.25E − 07	9032.366266	287.3015503	283965.1936
cg22280359	5.71E − 07	2038.684276	102.8744546	40401.02662
cg26206990	5.90E − 07	10087.35509	270.7518914	375822.7955
cg26780750	6.06E − 07	40844.3516	630.5307387	2645804.487
cg16188681	1.39E − 06	282.9628544	28.58738574	2800.814937
cg05492845	1.46E − 06	10101.82075	237.0738349	430443.041
cg06812522	1.48E − 06	481.591637	38.94307486	5955.628971
cg18908524	1.81E − 06	2309.260249	96.01135579	55542.21014
cg14358451	2.23E − 06	100451.6413	850.915178	11858446.64
cg15674575	2.37E − 06	1480.507671	71.39852872	30699.55365
cg13518339	2.75E − 06	12097.95681	237.7795786	615530.3993
cg21367923	3.64E − 06	4224.520817	123.3644837	144665.4305
cg01334818	3.66E − 06	35951.47558	423.7024981	3050509.739
cg23248865	4.04E − 06	3654.415779	111.6644816	119597.1584
cg26833830	4.25E − 06	31.44342533	7.23235369	136.7036291
cg15633416	4.54E − 06	15621.4833	251.823192	969055.8625
cg21582582	4.77E − 06	287.2274165	25.40824841	3246.960885

### Function enrichment analysis and construction of classifier

To identify the molecular type of gastric cancer based on methylation, we use QDMR software to identify specific methylation sites. For each cluster, calculate the average value of each methylation level in 22,062 methylation sites, and obtain the 22,062 × 6 matrix using the input data of QDMR software. Set the threshold to 0.15.

In order to observe the mechanism of action of these specific methylation sites, we annotated the corresponding genes in the promoter region where these specific methylation sites are located and used the Enrichr online tool to perform functional enrichment analysis to discover the functions of these gene enrichments. path. In order to verify the discrimination ability of the identified specific methylation sites, we further applied the 347 specific methylation sites identified by QDMR to construct a random forest classifier, and ten-fold cross-validation to determine the performance of the model.

## Results

### Uniform clustering block selection of molecular subtypes using single- and multi-factor significant methylation profiles

**Fig. 1:**
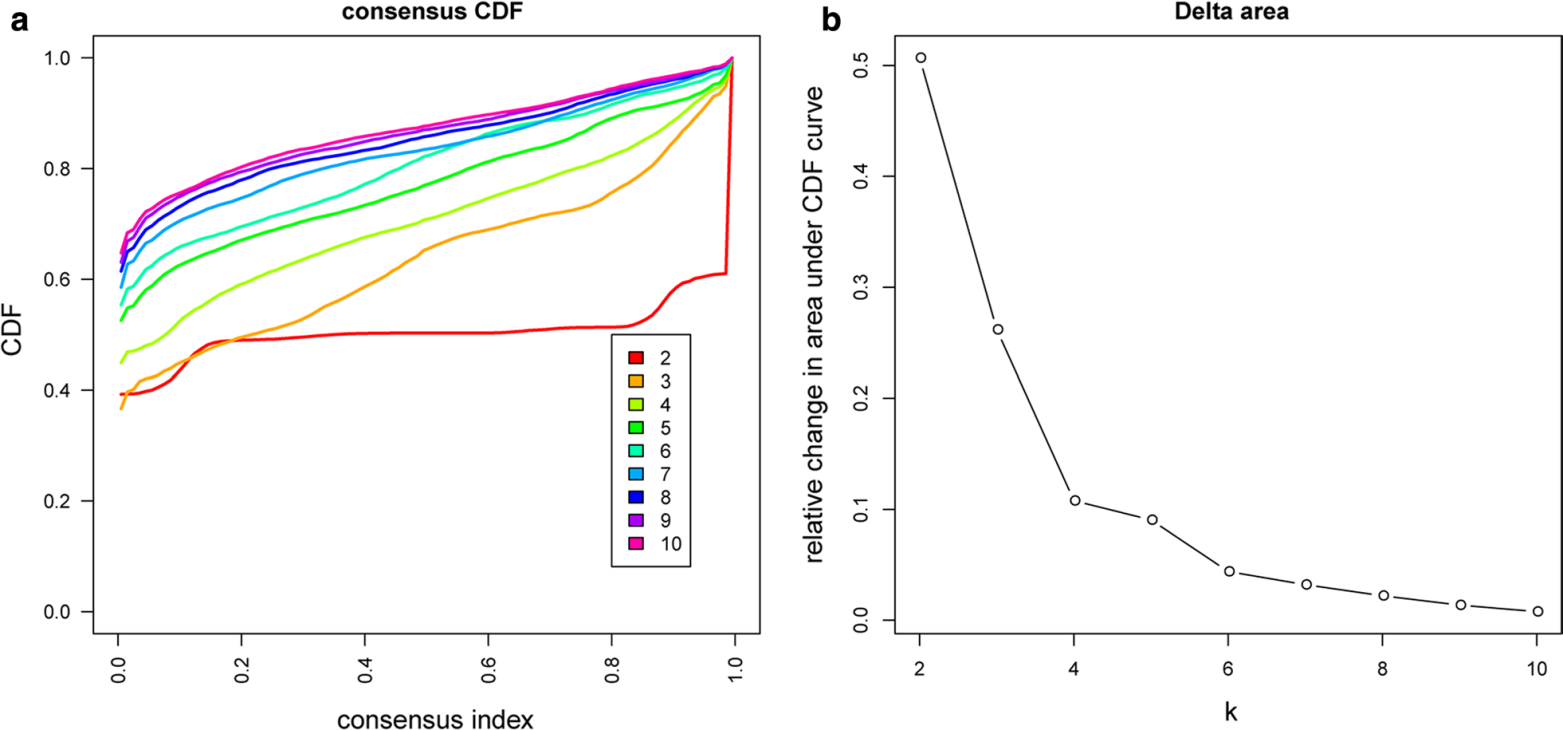
**a** CDF curve; **b** CDF Delta area curve

Using the resampling scheme to sample 80% of the samples and resampling 100 times, the optimal number of clusters is determined by the cumulative distribution function (CDF), as shown in Fig. 1a, from which we can see that Clusters have clustering at 5, 6 The results are relatively stable. Further observation of the CDF Delta area curve is shown in Fig. 1b. It can be seen that the clustering result is stable when Cluster is selected as 6, and finally we choose k = 6 to obtain 6 molecular subtypes.

### Cluster analysis of methylation expression profiles of six molecular subtypes

**Fig. 2:**
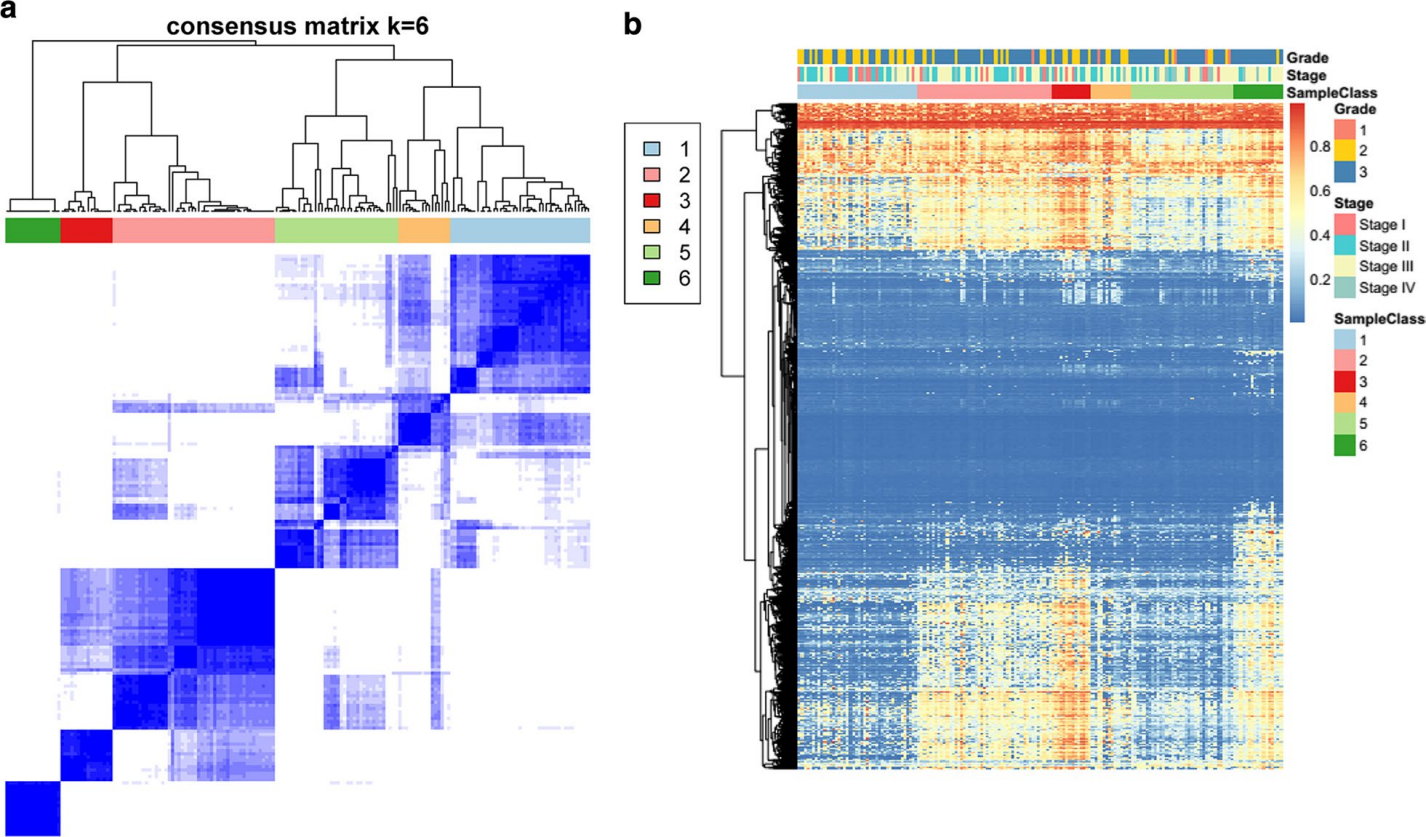
**a** Sample cluster heat map when consensus k = 6; **b** 22,062 methylation site clustering results in six types of samples

Based on the consistent clustering results, we selected a stable k = 6 clustering result as shown in Fig. 2a. It can be seen that tumor samples were assigned to these six categories. Further, we used 22,062 methylation spectra to perform Cluster analysis, using Euclidean distance to calculate the distance between methylation sites, as shown in Fig. 2b, it can be seen that most of the methylation sites are less abundant in each sample, while in the six categories There are also significant differences in methylation expression profiles, especially for Cluster1, which has significantly lower methylation levels than other types.

### Analysis of clinical characteristics of 6 molecular subtypes

**Fig. 3:**
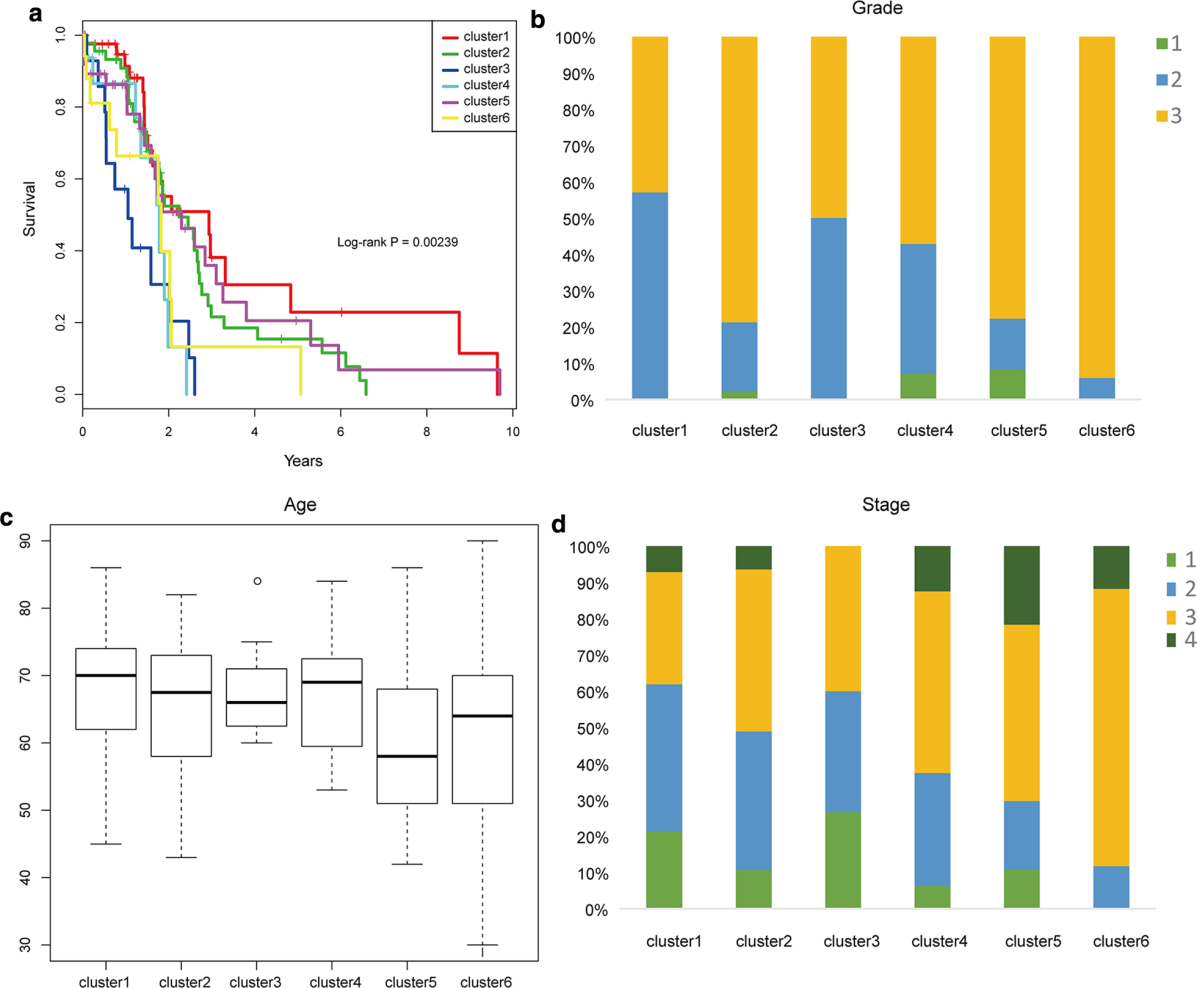
**a** Prognostic differences between six types of samples; **b** The proportion of different Grades in the six categories of samples; **c** age distribution in six categories of samples; **d** The proportion of different stages in the six categories of samples

Further, we analyzed the distribution of the prognosis, Stage, Grade, and Age of each sample in the six molecular subtypes as shown in Fig. 3, and it can be seen from Fig. 3a that there are significant prognostic differences among these six types of samples, among which Cluster1 has the best prognosis. Well, Cluster3 has the worst prognosis, which suggests that the prognosis of the hypo methylated sample is better than that of the hyper methylated sample. It can be seen from Fig. 3b that Cluster6 is associated with high invasion, and the age distribution of the six types of samples can be seen from Fig. 3c. From Fig. 3d, it can be seen that the patients in Cluster3 are associated with high Grade, and the higher patient’s stage, the shorter the survival time.

### Screening of specific methylation sites within the group

**Fig. 4:**
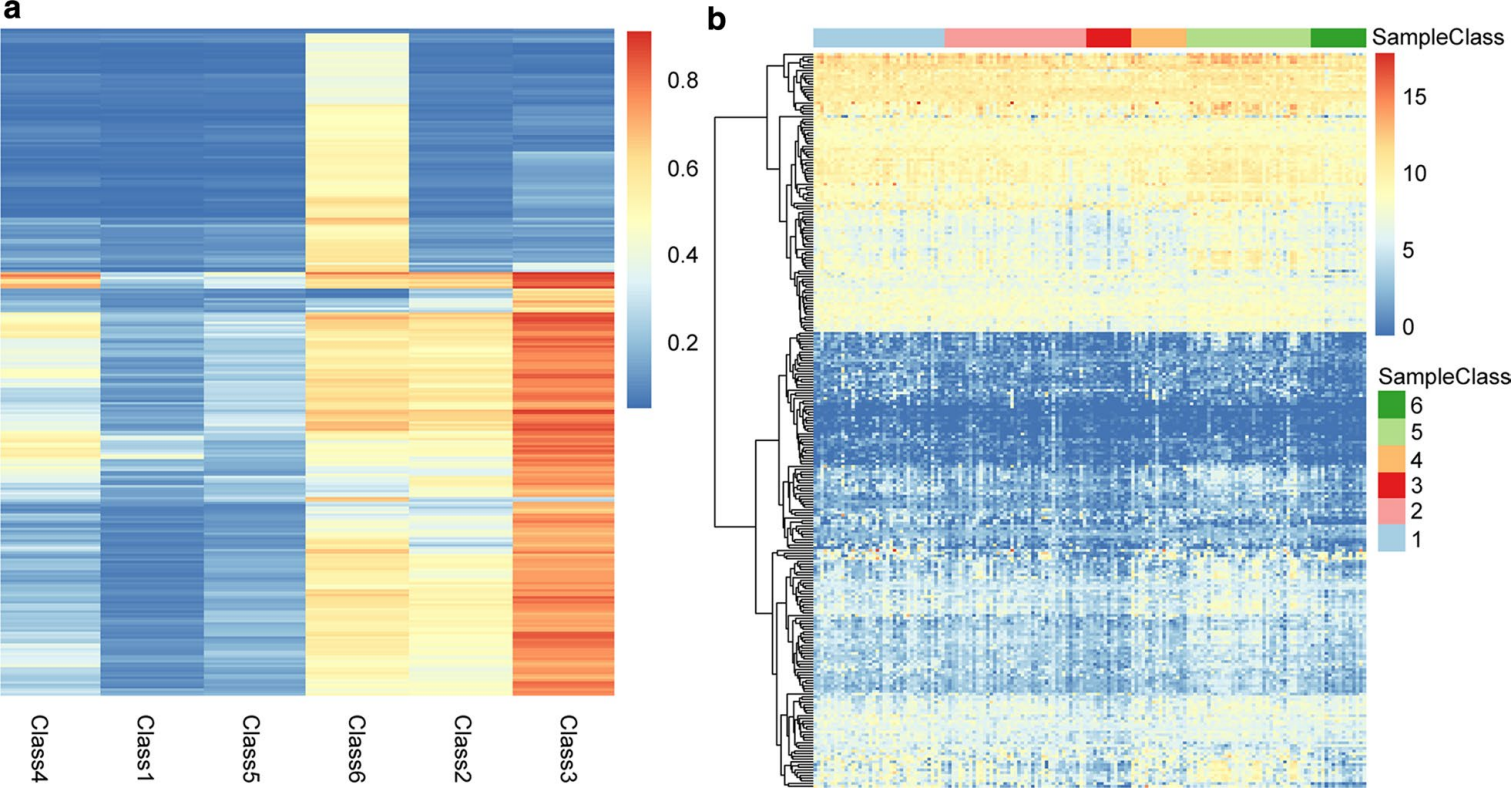
**a** distribution of methylation specific sites, **b** expression profile analysis of genes with specific methylation site annotations

Finally, 347 methylation sites that were considered to be cluster-specific methylation sites were screened, such as Additional file 2: Table S2. The heat map is shown in Fig. 4a. It can be seen that Cluster1 and Cluster3 have the most specific methylation sites. Cluster1 is mostly hypo methylated, Cluster3 is mostly hyper methylated, and there are a few specific methylation sites in other types. We annotate these 347 methylation sites by genomic annotation to obtain a total of 271 genes, such as Additional file 3: Table S3. In addition, we explored the gene expression of specific methylation sites in the subgroup, and found a total of 160 samples from the training set corresponding to the detected RNA-Seq. We extracted 271 genes of these 160 samples. The expression profile of the expressed genes, such as Additional file 4: Table S4, is further plotted as shown in Fig. 4b. It can be seen from the expression profile that these subgroups also have different expression patterns. This implies that there is a negative correlation between the DNA methylation level and gene expression.

### Functional enrichment analysis of genes with specific methylation site annotation

**Fig. 5:**
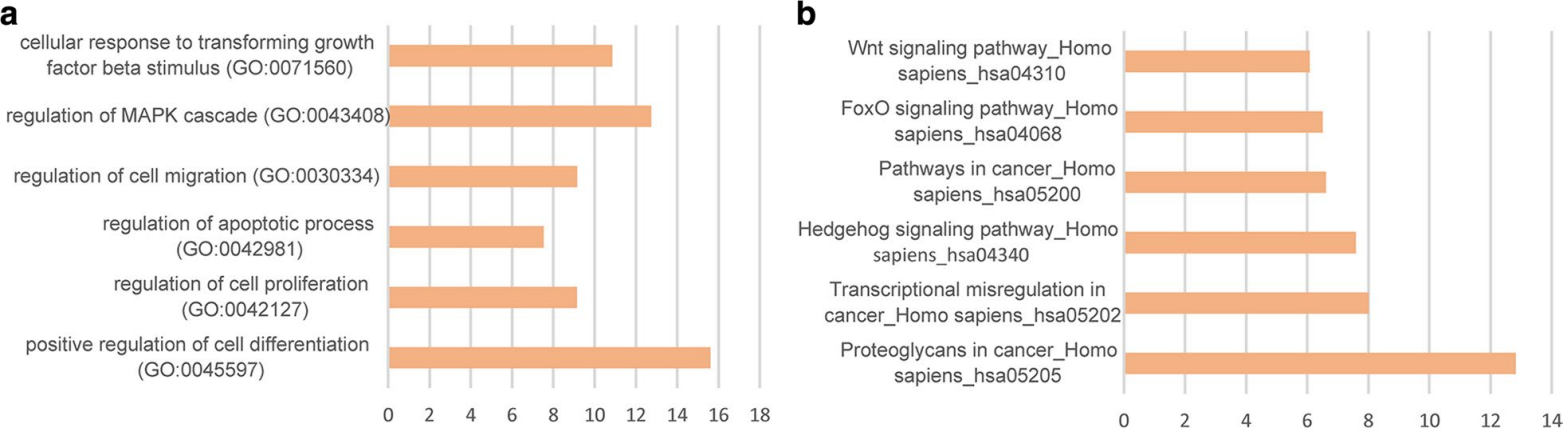
Functional enrichment results of specific methylation site annotation genes. **a** special genes are enriched in multiple cancer-related pathways; **b** special genes are enriched in multiple cancers of GO relevant biological processes

Through online tool enrichment analysis, we finally found that these genes were enriched in functional pathways related to gastric cancer. As shown in Fig. 5a, these genes are enriched in multiple cancer-related pathways of KEGG, including WNT signaling pathway, FoxO signaling pathway, cancer signaling pathway, and so on; as shown in Fig. 5b, these genes are enriched in multiple cancers of GO Relevant biological processes include cell response to transcription factor B, regulation of MAPK signaling pathways, and regulation of cell proliferation and metastasis. This indicates that the specific methylation probes identified in this study are closely related to gastric cancer.

### Construction of random forest classifier and data verification of independent test set

**Fig. 6:**
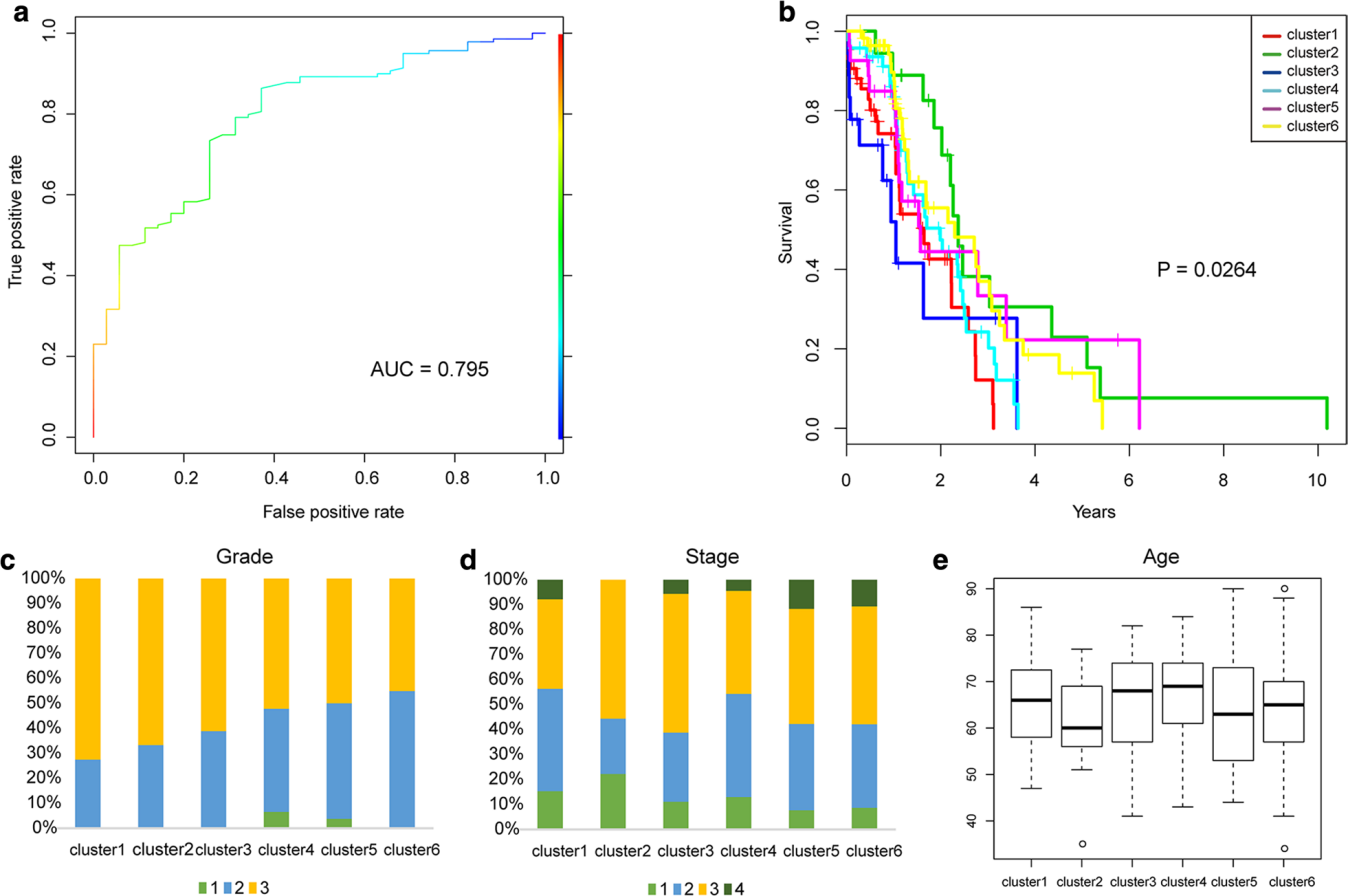
**a** AUC curve of the training set model; **b** predicted methylation pattern of the test set data after classification; **c** the proportion of different Grades in the six types of samples in the test set; **d** different stages of the six types of samples in the test set Proportional distribution; **e** Age distribution in six samples in the validation set

Through the construction of the random forest classifier, we found that the classification accuracy of the model based on the training set was 82.35%. The area under the ROC curve reaches 0.795, as shown in Fig. 6a. In order to verify the stability and reliability of the model, we extracted the expression profile data of the 347 CpG methylation sites (6 clusters) and substituted it into the test set to verify the model. The statistics of the prediction results are shown in Table 2. The number of categories of the sample and the training set are similar. Further analysis of the prognostic differences of the six types of samples is shown in Fig. 6b. It can be seen that there is also a significant difference in prognosis of these six types of samples, with a significant p value of 0.0264. The prognosis of the Cluster2 sample It is significantly better than other types of samples. Figure 6c shows the distribution of Grade in the test set sample, Fig. 6d shows the distribution of Stage in the test set sample, and Fig. 6e shows the distribution of patient age in the test set sample. The feature distributions of 6 types of samples in the test set and the training set are compared, and it is found that the three feature distributions have certain consistency. In short, the prognostic model constructed from these 347 methylation profiles has higher prediction accuracy and stability of the identified methylation features.

**Table 2:** Statistics of various samples predicted in the test set

Cluster	Number of samples
1	42
2	15
3	15
4	53
5	29
6	61

**Fig. 7:**
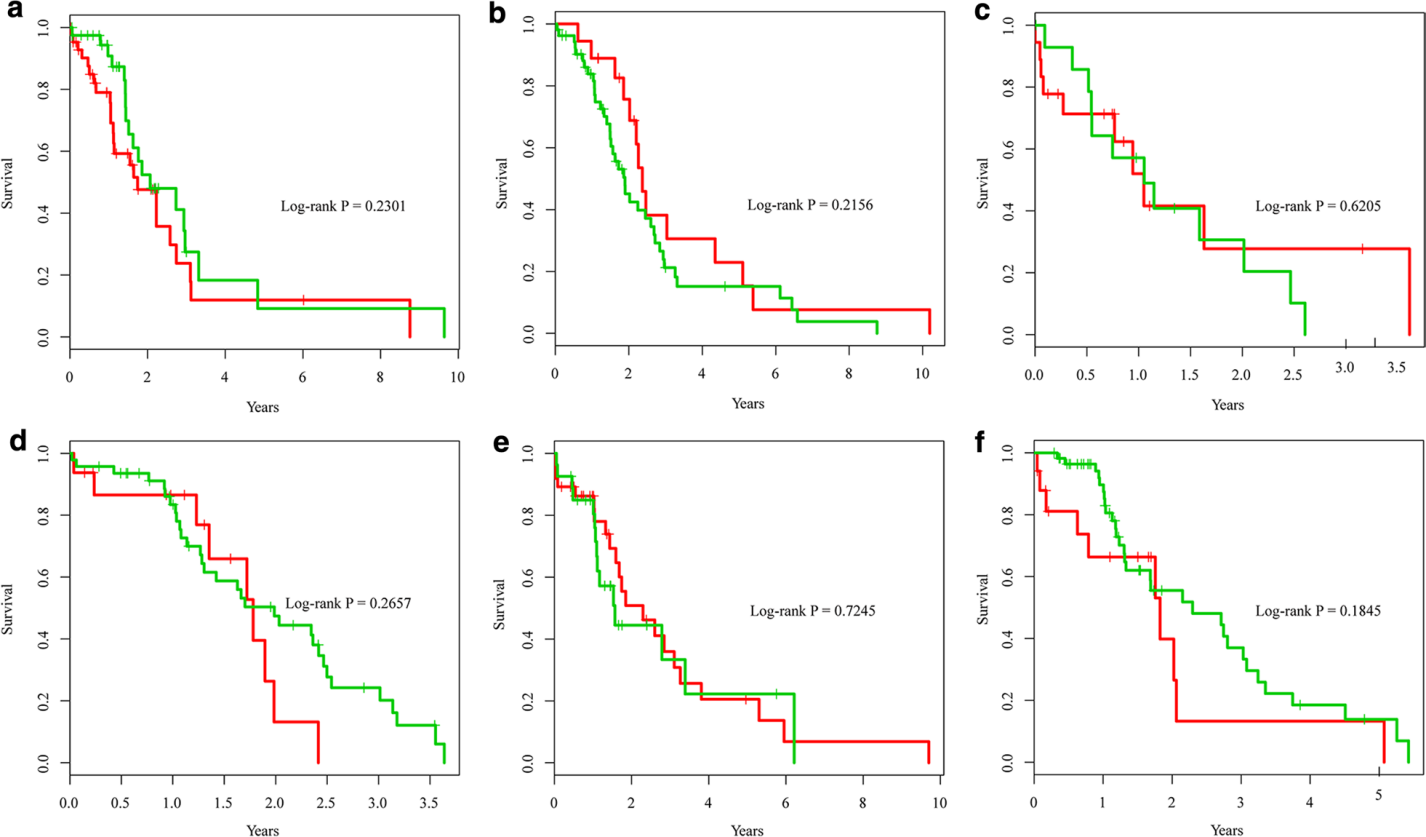
Difference of survival information between the training set and the test set of the 6 types of samples. **a–f** are the KM curves of cluster1 ~ cluster6 samples

In order to exclude the interference of the prediction results due to the particularity of the samples, this study compared the survival of the 6 types of patients in the training set and the test set. As shown in Fig. 7, the survival of the 6 types of samples in the training set and the test set were both Not significant, with a minimum P value of 0.1845 and greater than 0.05, which indicates that the methylation sites screened in this study can be applied to other gastric cancer samples and have certain potential significance in future research.

## Discussion

In the field of bioinformatics, the classification and prediction of gastric cancer has become an important subject. This method can explore the genesis and development mechanism of gastric cancer at the gene level, and can fundamentally study the cause of gastric cancer. The TCGA database collected epigenetic and transcriptome sequencing information for more than 30 cancers from thousands of patients [12]. Researchers began to explore the pathogenesis of related cancers at the genetic level, cancer genomics has become new directions for treatment [13–15].

Cancer classification models are one of the important components of cancer genomics research. Researchers usually combine various types of genetic sequencing data, such as DNA methylation, copy number variation, and original sequencing data, to explore precise cancer classification models and Cancer occurrence and development mechanism [16–19]. Most of the current literature researches are carried out by preprocessing operations such as data standardization, dimensionality reduction, and balance on various types of genetic sequencing data obtained [20]. Then, the pre-processed data set is input to the cancer classification model constructed in the study for learning and training, and the training parameters are continuously adjusted and the model is optimized during the model training process [21]. Finally, stable performance and strong generalization ability are obtained cancer classification system. The classification of gastric cancer has developed slowly, mainly because the early symptoms of gastric cancer are not obvious, and it is difficult to accurately classify them by pathological images. Therefore, this paper proposes to use the DNA methylation sequencing data of large-scale gastric cancer samples to learn the relevant characteristics of gastric cancer patients, and then construct and train a classification system suitable for gastric cancer, which provides a reference for the accurate early classification and diagnosis of gastric cancer.

This study combines gene expression profile data and DNA methylation data to pre-process missing values of public data, remove unstable CpG sites, and select methylation sites in the promoter region as the final methylation. Expression spectrum. Use the Consensus Cluster Plus package [22] to perform supervised cluster analysis on the data, select samples with both methylation and expression profile data, and build a training set for building a classifier to train the model or determine model parameters; the test set is used to The performance of the final selection of the best model is tested, and the generalized ability of the trained model is tested. In short, the data set is divided into two categories to prevent overfitting. Perform a single factor Cox analysis on each methylation site, Stage, Grade, and Age, select significant classification characteristics, further use M, Stage, Grade, and Age as covariates to introduce Cox, and further each significant methyl group Cox multivariate analysis was performed on the quantification sites, and significant methylation sites were screened as subsequent categorical variables. Through consistent cluster analysis of the expression profiles of these potential methylated biomarkers, we identified six molecular subtypes. By analyzing the prognostic differences of different molecular subtypes, we observed the prognosis of different molecular subtypes. Differences, while analyzing differences in clinical characteristics of different molecular subtypes. Using QDMR software analysis, we screened out specific methylation sites in these molecular subtypes. At the same time, we found 271 genes containing methylation sites for classification. What is more critical is that there is a large correlation between these 6 subclasses and clinical data and gene mutation data, suggesting that these 6 each subclass represents a different biological subtype. Enrichr, an online tool for gene enrichment analysis, was used to explore the molecular enrichment of tag-like genes to investigate the functional enrichment and related pathway enrichment of tag-like genes. Finally, we build a prognostic model of gastric cancer based on a random forest classifier model and model detection.

Gastric cancer can be classified and analyzed at different levels according to different molecular characteristics [23]. Identifying and identifying different subtypes of gastric cancer samples at these molecular levels can not only help people understand the disease at the root, but also help doctors choose the best medicine [24]. Treatment options predict the survival of different patients and identify high-risk factors associated with specific subtypes [25]. The purpose of this study is to expect to obtain gastric cancer subtypes that are related to gastric cancer biology and clinical data at the gene expression level and methylation level, and use the information gene to establish a classifier at the molecular level to predict the class assignment of new samples. In-depth gene enrichment analysis and co-expression network analysis of information genes were performed.

## Conclusion

This study was based on the TCGA methylation profile of gastric cancer to identify prognostic-specific methylation to construct a classifier for gastric cancer. Helps identify new molecular subtypes of gastric cancer. This classifier can provide guidance for clinicians on the diagnosis and prognosis of different epigenetic subtypes. In addition, the identified subtype-specific molecules provide multiple targets for the precise medical treatment of gastric cancer.

## Supplementary information

**Additional file 1: Table S1.** 22,062 significant methylation sites.

**Additional file 2: Table S2.** 347 cluster-specific methylation sites.

**Additional file 3: Table S3.** 271 genes obtained by genomic annotation.

**Additional file 4: Table S4.** The expression profile of the 271 genes in 160 samples.

## Data Availability

The supplementary data used and generated during the current study are available from the corresponding authors on reasonable request.

## Abbreviations

GC: Gastric cancer
TCGA: The cancer genome atlas
CDF: Cumulative distribution function
